# A randomised controlled trial investigating the causal role of the medial prefrontal cortex in mediating self-agency during speech monitoring and reality monitoring

**DOI:** 10.1038/s41598-024-55275-3

**Published:** 2024-03-01

**Authors:** Songyuan Tan, Yingxin Jia, Namasvi Jariwala, Zoey Zhang, Kurtis Brent, John Houde, Srikantan Nagarajan, Karuna Subramaniam

**Affiliations:** 1 Department of Psychiatry, University of California, 513 Parnassus Avenue, HSE604, San Francisco, CA 94143 USA; 2 Department of Psychology, Palo Alto University, Palo Alto, CA USA; 3 Department of Otolaryngology, University of California, San Francisco, San Francisco, CA USA; 4 Department of Radiology and Biomedical Imaging, University of California, San Francisco, San Francisco, CA USA

**Keywords:** Self-agency, Speech monitoring, Reality monitoring, Medial prefrontal cortex, Repetitive transcranial magnetic stimulation, Psychology, Cognitive neuroscience, Language

## Abstract

Self-agency is the awareness of being the agent of one's own thoughts and actions. Self-agency is essential for interacting with the outside world (reality-monitoring). The medial prefrontal cortex (mPFC) is thought to be one neural correlate of self-agency. We investigated whether mPFC activity can causally modulate self-agency on two different tasks of speech-monitoring and reality-monitoring. The experience of self-agency is thought to result from making reliable predictions about the expected outcomes of one’s own actions. This self-prediction ability is necessary for the encoding and memory retrieval of one’s own thoughts during reality-monitoring to enable accurate judgments of self-agency. This self-prediction ability is also necessary for speech-monitoring where speakers consistently compare auditory feedback (what we hear ourselves say) with what we expect to hear while speaking. In this study, 30 healthy participants are assigned to either 10 Hz repetitive transcranial magnetic stimulation (rTMS) to enhance mPFC excitability (N = 15) or 10 Hz rTMS targeting a distal temporoparietal site (N = 15). High-frequency rTMS to mPFC enhanced self-predictions during speech-monitoring that predicted improved self-agency judgments during reality-monitoring. This is the first study to provide robust evidence for mPFC underlying a causal role in self-agency, that results from the fundamental ability of improving self-predictions across two different tasks.

## Introduction

Self-agency is of cardinal importance because it underlies self-awareness in human interactions with the outside world (i.e., reality monitoring)^1^. Self-agency is thought to result from making reliable predictions about the expected outcomes of one’s own actions^1,2^. Compared to external actions, the sensory outcomes of self-generated actions are highly predictable^3–5^. This self-prediction ability is necessary for the accurate encoding and memory retrieval of one’s own thoughts during reality monitoring to enable accurate judgments of self-agency (i.e., accurate recognition of self-generated information)^6^. This self-prediction ability is also critical for feedback monitoring during speaking, where we are constantly comparing auditory feedback (i.e., what we hear ourselves say) with what we expect to hear when we speak^2^.

We and others have previously shown that activity within the medial prefrontal cortex (mPFC) mediates both lower-level self-predictions during speech monitoring^1,7^, and higher-level self-agency judgments during reality monitoring in healthy participants^6,8^. In our prior reality monitoring studies, in which subjects distinguish self-generated from externally-derived information, participants showed increased mPFC activity during the successful encoding and retrieval of self-generated information, which correlated with accurate judgments of self-agency, indicating mPFC likely represents an early neural correlate of self-agency^6,8^. In our speech monitoring tasks, subjects experience self-agency only when auditory feedback slightly deviates from predictions of what they expect to hear^1,2^. When subjects hear such small perturbations in their auditory feedback while speaking, they make corrective responses, indicating that they judge the perturbations as errors in their speech output^2,3,9,10^. The peak magnitude of these corrective responses is modulated by subjects’ reliance on self-predictions about their speech outcome,the more they rely on their self-predictions, the less they rely on perturbed external auditory feedback, resulting in smaller corrections and an enhanced sense of self-agency that they followed their self-predictions to guide their own speech output. In our prior speech monitoring studies, participants also showed increased mPFC activity when they made smaller corrections to pitch perturbations in their auditory feedback, indicating greater reliance on self-predictions of their expected speech outcome^1,7^.

The mPFC represents one region that replicably shows increased activity during self-predictions prior to self-generated actions (that do not occur before external actions), across convergent evidence from functional imaging (functional magnetic resonance imaging (fMRI), magnetoencephalography (MEG), electroencephalography (EEG)) and single neuron studies^6,8,11–15^, thus indicating that mPFC likely represents one neural site that mediates the self-prediction computations that results in the experience of self-agency. Given these correlative data that mPFC supports both self-predictions and self-agency in healthy participants, we now test whether mPFC activity can causally modulate this self-prediction ability to impact self-agency on two different tasks of reality and speech monitoring. This will show that mPFC provides a unitary basis for self-agency, driven by reliance on self-predictions. Here, we use repetitive transcranial magnetic stimulation (rTMS) as a causal neurostimulation tool to test whether increasing mPFC excitability with high-frequency 10 Hz rTMS, will enhance self-predictions during speech monitoring to predict better self-agency judgments during reality monitoring. Specifically, in the present study, we implemented a subject-blinded randomized controlled trial (RCT) in which participants are assigned to either active rTMS to enhance mPFC excitability or 10 Hz rTMS applied to the left temporoparietal site (N = 15). Using reality and speech monitoring tasks, measured from pre-to-post rTMS, we examined the causal mechanisms of how active rTMS modulation of mPFC activity induces changes in the self-agency network in healthy participants, compared to baseline and temporoparietal rTMS.

We had three specific hypotheses: (1) Compared to temporoparietal rTMS, enhancing mPFC excitability by active 10 Hz rTMS would induce participants to make smaller corrective responses during minimal pitch-induced perturbations. In other words, we predicted that speakers would make smaller corrective responses during these minimal pitch-induced perturbations after rTMS to mPFC, and this would reflect their increased reliance on internal self-predictions to guide their speech output, rather than reliance on external altered feedback to influence their speech output, which would reflect their enhanced sense of self-agency. (2) Compared to temporoparietal rTMS, enhancing mPFC excitability by active 10 Hz rTMS would improve self-agency judgments (i.e., improved accurate identification of self-generated information) while participants perform the reality monitoring task. (3) Smaller corrective responses induced by rTMS applied to mPFC would predict improved self-agency judgments during the reality monitoring task. If these hypotheses are confirmed, the present study would provide the first causal evidence for mPFC activity underlying a unitary sense of self-agency that is driven by the basic ability of making reliable self-predictions during a speech monitoring task, which potentiates self-agency judgments on a reality monitoring task.

## Methods

### Participants and procedures

In the present subject-blinded randomized controlled trial (RCT), 30 healthy participants (20 males, 10 females, mean age = 43 years, mean education = 17 years) volunteered to participate in this study at the University of California San Francisco (UCSF). This study was approved by the Internal Review Board (IRB) at UCSF. All procedures were performed in accordance with NIMH and IRB guidelines at UCSF. Participants were recruited through our clinicaltrial.gov site (NCT04807530) or from our previous research studies if they had consented to be contacted for future studies. Participants were evaluated by a clinical psychologist and completed questionnaires to meet the inclusion criteria. Inclusion criteria for healthy participants were absence of neurologic psychiatric disorders [Axis I or Axis II (SCID-Nonpatient edition)] and major illnesses, no current or history of substance dependence or abuse, meets MRI criteria, good general physical health, age between 18 and 64 years, right-handed, and English as the first language. All participants provided written informed consent for this study and then completed structural magnetic resonance imaging (MRI), a speech-monitoring task and a reality-monitoring task at baseline. Subjects were matched at a group level on age, gender, and education, and then randomly assigned in a subject-blinded RCT to either the active 10 Hz rTMS condition targeting mPFC (n = 15), or the temporoparietal 10 Hz rTMS condition (n = 15) (Table 1). One participant who was assigned to the active rTMS condition was not available to complete the post-rTMS assessments. Immediately after the rTMS session, all participants completed the speech-monitoring and reality-monitoring tasks.

**Table 1:** Demographics (mean, SD) of healthy participants.

	Subjects in rTMS to mPFC	Subjects in rTMS to left temporoparietal site	P value
Age (years)	41 (16)	44 (18)	0.60
Gender	10 M, 5 F	10 M, 5 F	1.0
Education (years)	17 (2.2)	17 (1.8)	0.86

### Speech monitoring task

Participants performed a speech monitoring task at baseline and immediately after the rTMS session. Participants wore a microphone (AKG Pro Audio C520 Professional Head-Worn Condenser Microphone, AKG Acoustics, Vienna, Austria) and a pair of headphones. The microphone was attached to an amplifier that was connected to a Dell computer sound card (M-Audio Delta 44 4 × 4 analog I/O, M-Audio, Cumberland, RI). The amplified audio signal was played back via the headphones. Participants confirmed they could clearly hear the audio signal prior to the experiment.

The speech-monitoring pitch perturbation experiment was programmed in Matlab, and consisted of 9 runs constituting 15 trials per run, totaling 135 trials. Each trial began with a green dot that appeared on the computer screen. Participants were instructed to vocalize the vowel /ɑ/ when they saw the green dot. They continued phonation for 2.5 s until the dot disappeared while listening to real-time auditory feedback from the headphones. In each trial, the phonation onset triggered a brief perturbation (of 100 cents or 1/12th of an octave) in the pitch of each participant’s auditory feedback^1,2,16,17^ for a duration of 400 ms following a variable delay between 200 and 500 ms from phonation onset. The direction of the pitch shift was randomized so that it was either upward or downward so that half the trials had a positive shift and the other half had a negative shift. This jittered perturbation and pseudo-random distribution minimized expectation/anticipatory bias, preventing participants from being able to predict either the onset or direction of the pitch shift. The inter-trial interval was 2.5 s during which time participants viewed a blank screen.

**Figure 1:**
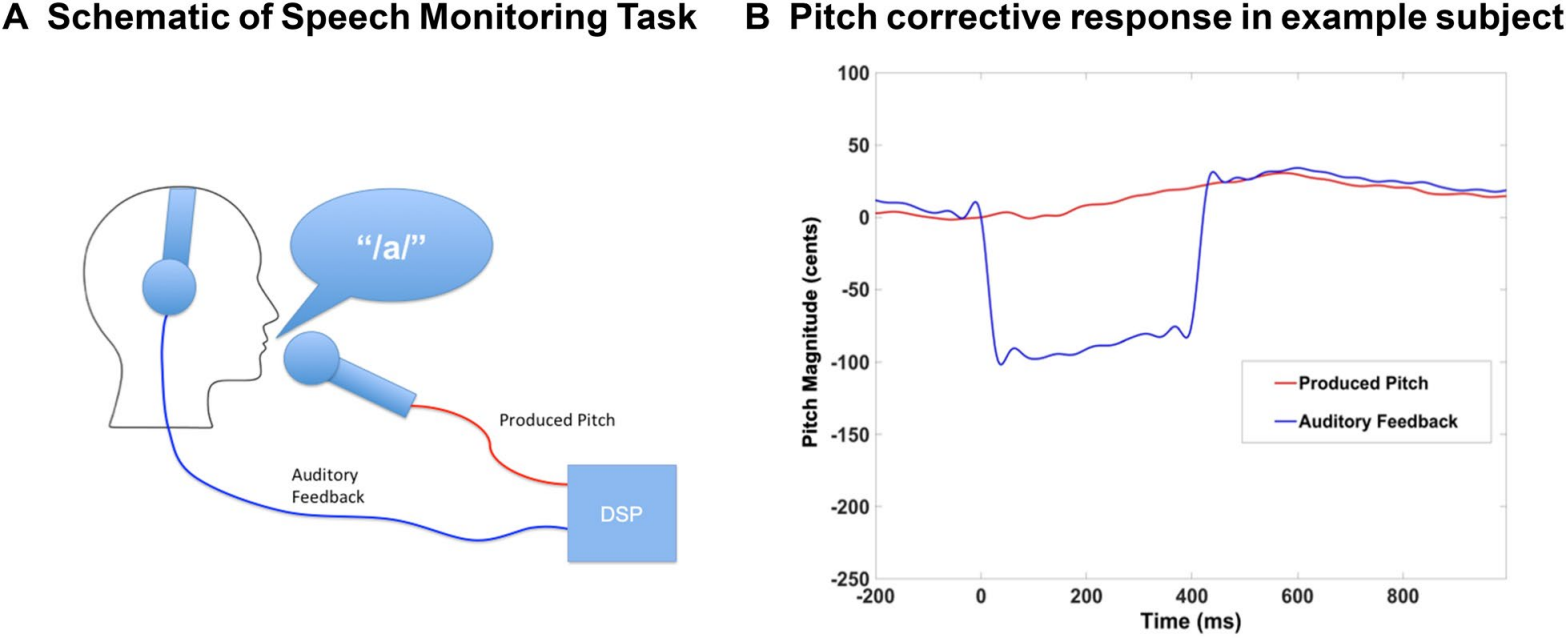
**(A)** During the speech monitoring task, participants produced a steady-state /a/ vowel. Their speech was picked up by a microphone and passed through a digital signal processing (DSP) program that provided auditory feedback of their speech in real-time that they heard in the headphones. In each trial of the experiment, the DSP was directed to alter the pitch of the participant’s speech feedback by ± 100 cents. (**B**) Example of one trial from one participant, in which the participant raised their pitch to partly compensate for the effects of the DSP which perturbed the participant’s vocal feedback by lowering the pitch for 400 ms by 100 cents.

Pitch perturbation was achieved by utilizing a real-time speech feedback alteration technique implemented by a digital signal processing (DSP) program (see also Raharjo et al.^18^) (Fig. 1A, B). The DSP program received the participant's vocalization as input, which was captured by an optical microphone (Phone-Or Ltd., Or-Yehuda, Israel). The output generated by the DSP program was then delivered back to the participant through earphones (model EAR-3A, Etymotic Research, Inc., Elk Grove Village, IL). The DSP program employed a vocoder process that decomposed incoming speech into pitch and spectral envelope characteristics, and either raised or lowered the pitch of each participant’s outgoing speech in real-time by 100 cents.

### Speech monitoring pitch perturbation analyses

For each participant and for each trial, the raw audio data were examined to extract the pitch time course using an autocorrelation-based pitch tracking method (Parsons, T.W. 1987. Voice and Speech Processing. Mcgraw-Hill College, Blacklick, OH). Participants who had noisy data with pitch tracking errors or incomplete utterances within the analysis interval were excluded (n = 3 from the active mPFC rTMS condition, and n = 2 from the temporoparietal rTMS condition). An analysis interval of 1200 ms was extracted for each trial, which spanned from −200 to 1000 ms relative to the perturbation onset, and was converted from hertz to cents, indicating the deviation from the pre-perturbation baseline. For each participant, the pitch track of each trial was processed by calculating the deviation from the mean pitch track, which was averaged across all trials, encompassing both upward and downward pitch perturbations. The responses to both upward and downward perturbations were combined into a unified dataset representing the absolute magnitude of the response for each participant. Participants responded to applied pitch perturbations by making corrective responses and deviating from their baseline pitch track. For each trial we computed the magnitude of pitch deviations in each participant, by calculating the peak corrective response with respect to the baseline pitch value prior to perturbation. For each participant, we then extracted the mean peak response across trials, as we have performed in our prior studies^1–3,17^. Compensatory corrective responses were defined as a mean pitch peak response which opposed the direction of the applied pitch perturbation shift, and yielded positive values. In other words, if the pitch shift was upward, and the response was downward, this denotes a positive compensatory response. Similarly, if the pitch shift was downward and the response was upward, this also denotes a positive compensatory response. The peak magnitude of the corrective responses was pooled together across the upward pitch perturbation shift trials and downward pitch perturbation shift trials. Consistent with our earlier studies^2,17,19^, these analyses techniques examined response deviations from the mean response track and thus the magnitudes of vocal responses to both the upward and downward pitch feedback perturbations were the same distance from baseline and did not change as a function of stimulus direction.

### Reality monitoring task

**Figure 2:**
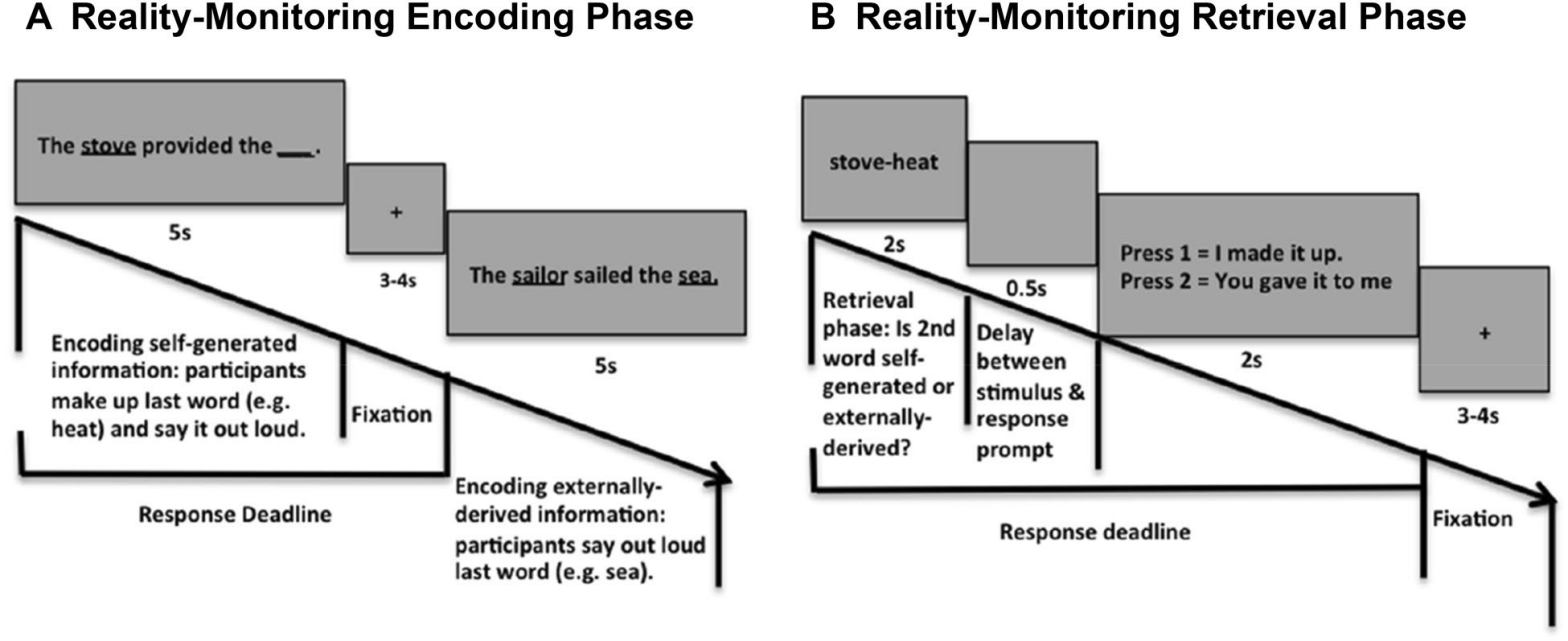
Reality monitoring task design. (**A**) During the encoding phase, participants were given sentences in which the final word was either left blank for participants to generate themselves (e.g., the stove provided the __) or was externally-derived as it was provided by the experimenter (e.g., The sailor sailed the sea). (**B**) During the retrieval phase, participants were randomly presented with the noun pairs from the sentences (e.g., stove-heat), and had to identify with a button-press whether the second word was previously self-generated or externally-derived.

Participants in the study performed a reality monitoring task at baseline and immediately after the rTMS session. As described in previous studies^1,2,8,20^, the reality monitoring task consisted of an encoding phase and a memory retrieval phase (see also Fig. 2A, B). All participants completed eight runs, with 20 trials per run, totaling 160 trials for the whole task. During the encoding phase, participants were visually presented with semantically-constrained sentences with “noun–verb-noun” structures. On half of the sentences, the final word was either left blank for participants to make up themselves (e.g., *The stove provided the* ), or was externally-provided by the experimenter (e.g., *The sailor sailed the sea*) (Fig. 2A). For each sentence, participants were told to pay attention to the underlined nouns for a subsequent memory test and to vocalize only the final word of each sentence. After the encoding phase, participants then completed the memory retrieval phase where they were randomly presented with the underlined noun pairs from the sentences (e.g., *stove-heat*), and were asked to identify whether the second word was previously self-generated or externally-derived using a button box. At each time-point (i.e., baseline and after rTMS), the sentences were completely different, containing different sets of matched semantically-constrained sentences.

### Reality monitoring analyses

For each participant, self-agency accuracy was computed as the percentage of correctly-identified self-generated items out of the total number of self-generated trials on the reality monitoring task. Signal detection theoretic d-prime analyses were performed to compute overall reality monitoring accuracy. This was done by calculating the hit rate and the false alarm rate for self-generated and externally-presented items, then converting each measure to z-scores, and subtracting the false alarm rate from the hit rate in order to differentiate sensitivity during accurate performance from response bias.

### rTMS protocol

Our TMS system is a state-of-the-art Nexstim Neuronavigated Brain Stimulator (Helsinki, Finland). This system integrates the TMS figure-8 coil with a navigational system with several landmarks on each subject’s scalp surface matched to their anatomical locations on the 3D MRI that allows for highly accurate cortical targeting individualized to each subject’s MRI anatomy. Once the targeted stimulation site is selected, the system calculates the strength of the electric field in real-time that is incident on the targeted cortical site^21,22^. The specific site that we target in subjects assigned to the active rTMS condition (MNI: x, y, z = −16, 48, 6), is based on our prior functional localization of medial frontal peak activity mediating self-agency during reality monitoring^8^. We have previously shown that this site can be directly modulated with rTMS to induce significant improvement in self-agency judgments during reality-monitoring^20^. This mPFC site has consistently shown increased activation during the successful encoding and retrieval of self-generated information, which correlated with self-agency judgments, across fMRI and MEG methodologies^6,8^. In these prior studies, we did not find activation of left posterior temporoparietal site underlying self-agency^6,8^, and thus considered this site to be outside the self-agency network. We selected the left posterior temporoparietal MNI coordinates (x, y, z = −50, −41, 38), as this site has been shown to be activated in our prior studies and others when speech output and motor actions are more likely to be experienced as external perturbations, and thus reflects external agency^16,23^. This site, therefore, provides a good control site, representing the dissociation between self-agency mediated by the mPFC and external agency, mediated by the temporoparietal site.

In our previous study^20^, we had established the optimal rTMS dosage parameters that maximized tolerability/comfort in the present study. The present rTMS parameters have also been deemed safe by the rTMS Consensus Guidelines^24^. The rTMS session consisted of 120 trains of 20 pulses (2 s duration of 10 Hz) for 20 min at 110% Resting Motor Threshold based on hand electromyography, which we have previously shown to maximize safety and efficacy, without a single adverse event^20^. Due to prior reports of more discomfort and pain with higher-frequencies from our prior pilot study we did not use frequencies of more than 10 Hz or theta-burst stimulation, or longer protocols (single train durations > 2 s).

### Statistical analyses

Repeated-measures ANOVAs were implemented to examine group differences, after rTMS compared to baseline, in peak deviation during the 100 cents pitch perturbations, as well as in self-agency judgments (i.e., self-generated identification accuracy) and overall reality monitoring performance (d-prime score). Pearson’s two-tailed correlation tests were used to measure the strength of the linear relationship between peak deviation for 100 cents pitch shifts with self-agency judgments and reality-monitoring performance. Effect sizes (Cohen’s d) were used to quantify the power of the group differences and linear relationships.

## Results

**Figure 3:**
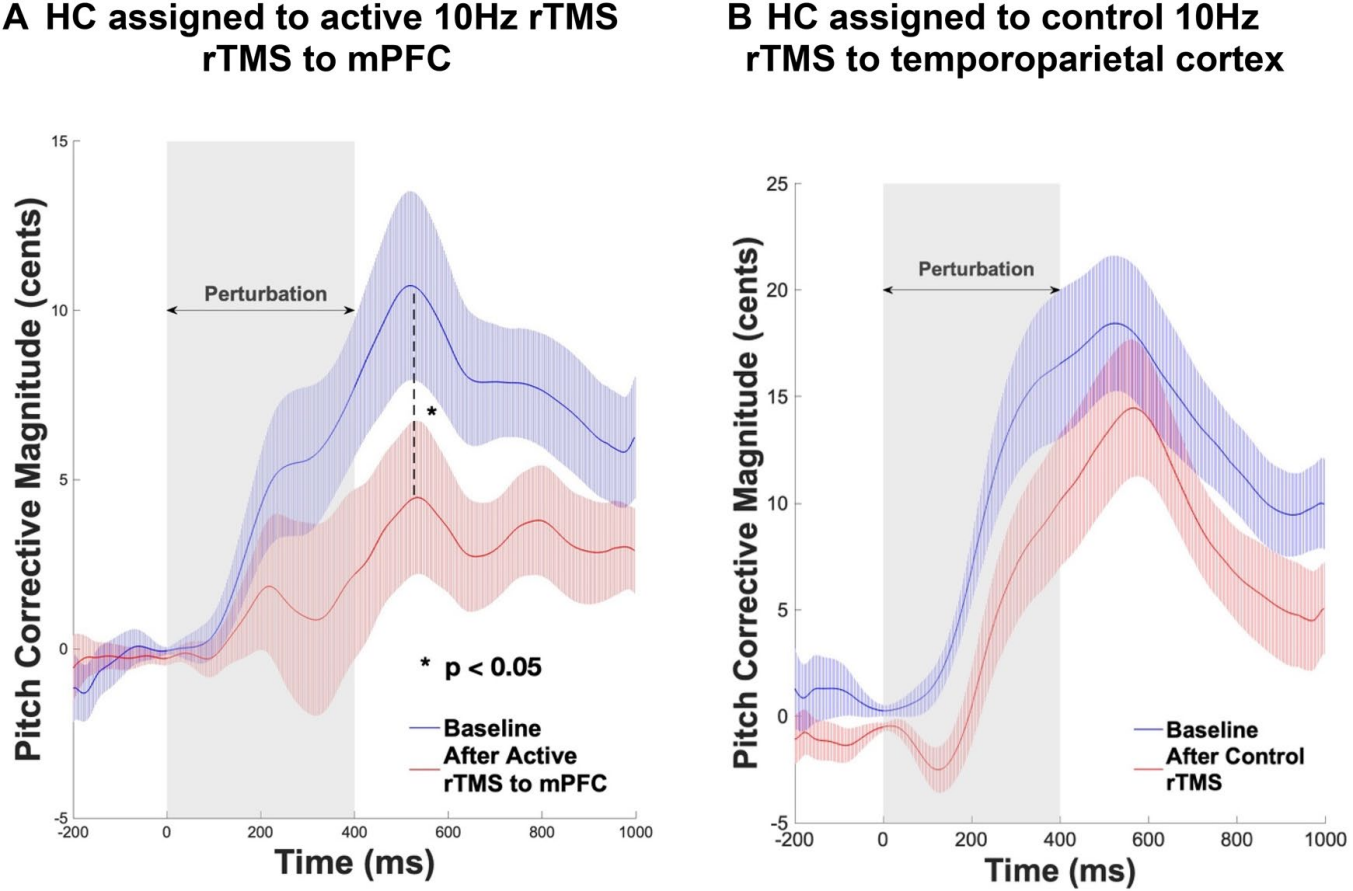
Mean pitch magnitude corrective responses during 100 cents pitch shifts, averaged across all participants at each time-point (i.e., baseline and after rTMS) in each condition shown for: (**A**) participants assigned to active rTMS applied to mPFC and (**B**) participants assigned to rTMS applied to left temporoparietal cortex. The grey rectangle illustrates the 400 ms duration of the experiment-induced pitched perturbation. Participants began responding to the applied perturbation at 117 ms after perturbation onset and peak response was attained at 536 ms after perturbation. Dashed lines represent the standard error of the corrective responses across all trials and participants for each condition and time-point.

Participants generated corrective behavioral responses to the 100 cents pitch perturbations in auditory feedback, most of which opposed the direction of the applied shift. On average, participants started responding to the applied perturbation at 117 ms after perturbation onset and peak response was reached at 536 ms after perturbation (Fig. 3). Consistent with our hypothesis, compared to baseline and the temporoparietal rTMS condition, enhancing mPFC excitability by rTMS induced participants to make significantly smaller corrective peak responses to the 100 cents pitch shift (F = 5.77, p = 0.04 and F = 8.58, p = 0.01, respectively) (Fig. 3). We did not find any difference in peak corrective responses after rTMS applied to the temporoparietal site compared to baseline (F = 1.2, p = 0.29). We also did not find any difference between the active rTMS and the temporoparietal rTMS conditions in the time to reach peak magnitude from perturbation onset either at baseline or after rTMS (all p’s > 0.05) (Fig. 3A, B).

**Figure 4:**
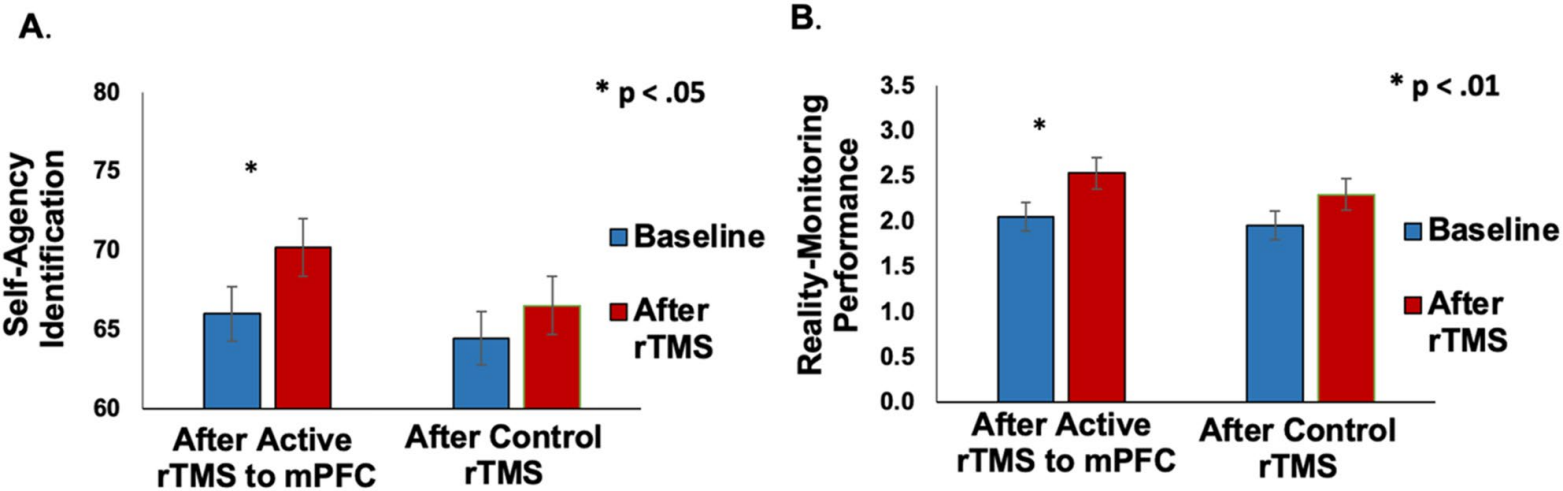
(**A**) Repeated-measures ANOVA revealed participants had significant improvement in self-agency judgments (i.e., % accuracy for self-generated information) that was observed only after they completed active rTMS to mPFC compared to baseline, but not for participants who completed rTMS to the left temporoparietal site. (**B**) Repeated-measures ANOVA revealed participants had significant improvement in overall reality monitoring performance (indexed by the d-prime score) that was also observed only after they completed active rTMS to mPFC compared to baseline, but not for participants who completed rTMS applied to left temporoparietal cortex.

Consistent with our hypothesis, on the reality monitoring task, enhancing mPFC excitability by rTMS induced significant improvement in self-agency judgments (F = 7.7, p = 0.02), which contributed to improved reality monitoring performance (F = 14.1, p < 0.01) (Fig. 4A, B). We did not find any difference after rTMS to the left temporoparietal site compared to baseline, in either self-agency judgments or reality monitoring performance (all p’s > 0.05). We also did not find significant group by time interactions in either self-agency judgments on the reality monitoring task or corrective peak responses in the speech monitoring task in this relatively small sample of healthy participants (all p’s > 0.05).

**Figure 5:**
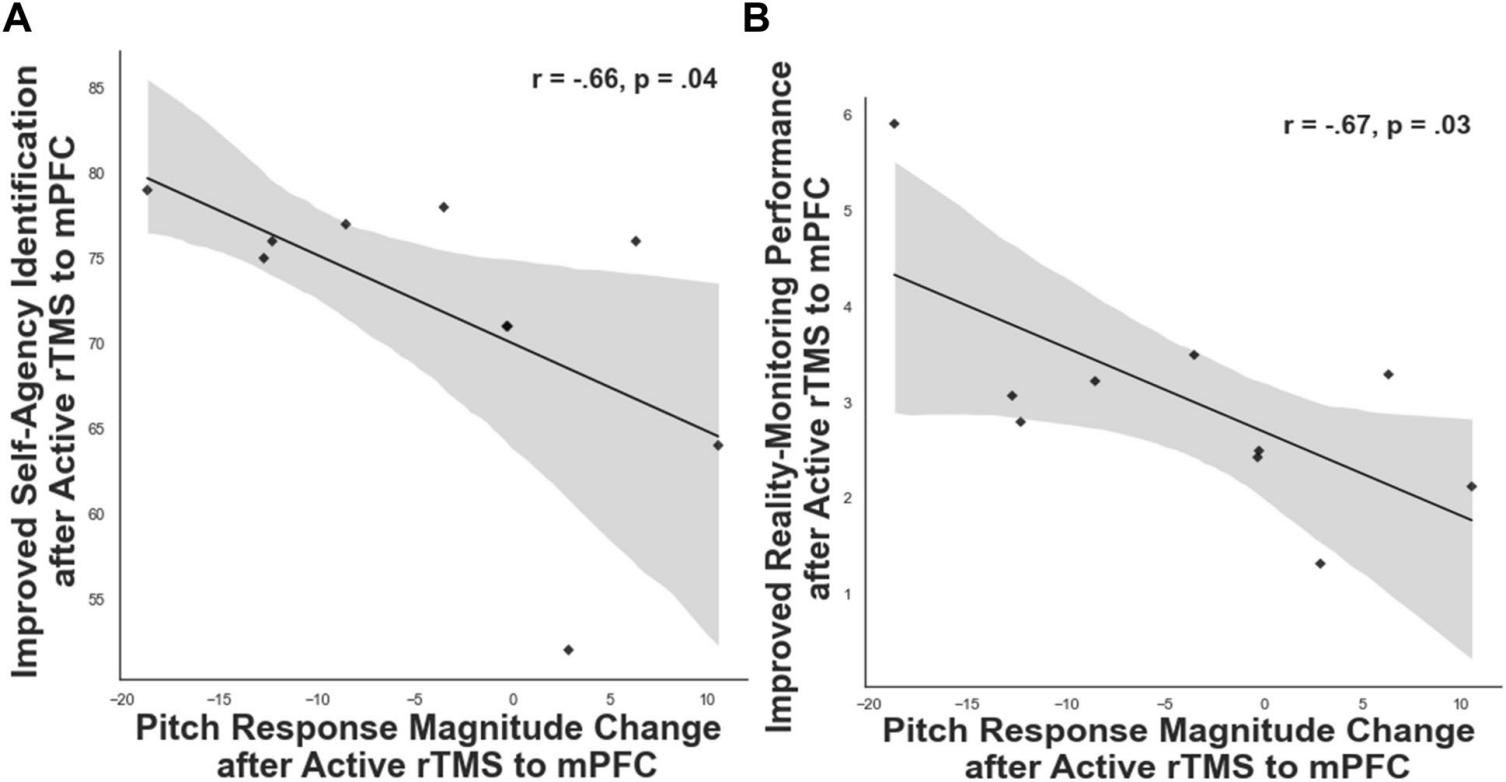
(**A**) The scatterplot illustrates the significant negative correlation between the smaller change in peak corrective response to 100 cents pitch perturbations induced by 10 Hz rTMS to mPFC with improved self-agency judgments (i.e., % accuracy for self-generated information) that was observed only after participants completed active rTMS to mPFC, but not for participants who completed rTMS applied to left temporoparietal cortex. (**B**) The scatterplot illustrates the significant negative correlation between the smaller change in peak corrective response to 100 cents pitch perturbations induced by 10 Hz rTMS to mPFC with improved reality monitoring performance (indexed by the d-prime score) that was observed only after participants completed active rTMS to mPFC, but not for participants who completed rTMS applied to the left temporoparietal cortex.

However, as confirmed by our a priori hypotheses, we found a significant negative correlation between peak corrective responses to the 100 cents pitch perturbations with self-agency judgments (r = −0.66, p = 0.04) and with reality-monitoring performance (r = −0.67, p = 0.03) (Fig. 5A, B). In other words, participants who made reduced corrective responses to compensate for their errors during pitch-induced perturbations demonstrated a greater sense of self-agency, which also potentiated their overall reality monitoring performance.

Overall, these findings replicate and extend our prior findings^1,2,20^ and provide a causal basis in delineating the mechanism for how enhancing mPFC excitability by rTMS increased participants’ reliance on their internal self-predictions to guide their speech output, rather than reliance on external altered feedback to influence their speech output, which predicted and potentiated improved self-agency judgments on the reality-monitoring task.

We did not find any associations between pitch perturbation corrective responses and accuracy for externally-derived information on the reality monitoring task (p > 0.05). These results replicate and extend our prior results^1,2^, in which we expected to only find correlations between self-agency judgments during reality monitoring with the peak magnitude of corrective responses during minimal pitch-induced perturbations experienced with the 100 cents pitch shift, as only these minimal perturbations are thought to maintain the sense of self-agency. Additionally, the time to reach peak corrective response was not associated with agency judgments during reality monitoring, revealing that only the peak magnitude of corrective responses provided reliable markers of self-agency judgments during the 100 cents pitch perturbation shifts (all p’s > 0.05). Finally, after rTMS to mPFC compared to baseline, Cohen’s d analyses yielded large effect sizes showing: (1) subjects made smaller pitch corrective responses, (2) subjects manifested improved self-agency judgements and (3) subjects manifested improved reality-monitoring performance (Cohen’s d = 0.73, 0.74, 0.72, respectively), as well as showing large effect sizes for the (4) correlation coefficients between rTMS-induced improved self-agency judgments and reality-monitoring performance with peak corrective responses during the 100 cents shift (Cohen’s d = 0.44 and 0.45, respectively). Together, these convergent findings across different types of analyses provide causal evidence for our hypothesis that the mPFC mediates the mechanisms underlying a unitary sense of self-agency that results from the ability to make *reliable* internal self-predictions about the outcomes of self-generated actions.

## Discussion

In this subject-blinded RCT we found that, compared to left temporoparietal rTMS, enhancing mPFC excitability by 10 Hz rTMS induced subjects to: (1) make significantly smaller corrective responses to the 100 cents pitch shifts, (2) improve self-agency judgments and overall reality-monitoring performance, (3) manifest a significant association between smaller corrective responses on the speech monitoring task that predicted improved self-agency judgments during the reality monitoring task. The present findings were only observed for participants who completed active 10 Hz rTMS targeting the mPFC, and were not observed in the temporoparietal rTMS condition. Together, these findings indicate that the improvements in self-agency across both speech monitoring and reality monitoring tasks were specific to rTMS modulation of mPFC excitability, and cannot be attributed to practice task effects or general effects of rTMS.

Our findings are consistent with our prior studies in which we have previously shown a significant association in healthy subjects who made smaller pitch corrective responses on the speech monitoring task who also manifested a greater sense of self-agency on the reality monitoring task^1,2^. Here, we now extend these prior correlative studies to delineate the causal mechanisms for how enhancing mPFC excitability by rTMS induced participants to make smaller corrective responses, reflecting their increased reliance on self-predictions to guide their speech output, which predicted and potentiated improved self-agency judgments on the reality-monitoring task.

Our results are also consistent with prior research indicating that subjects would produce corrective responses to oppose the error in their speech output that arises from the difference between the perceived and predicted auditory feedback (i.e., the prediction error), especially during minimal 100 cents pitch perturbations, as only these minimal pitch perturbations are thought to maintain the sense of self-agency^25–27^. For example, participants are able to consciously judge larger pitch perturbations greater than 250 cents as non self-generated outcomes that are due to external changes in the environment, rather than being indicative of errors in their own speech output (i.e., self-agency)^25–28^. The sensory outcome of self-generated actions is well-predicted, compared to external actions^3,4^. Thus, typically the smaller the prediction error between the actual and predicted auditory feedback, the more likely the outcome will be attributed as a self-generated. However, we also believe that the sense of self-agency is not a direct result of this minimal prediction error. Rather, we believe that the mPFC produces an estimation of the ‘amount of reliance’ or the gain that needs to be placed on self-predictions and the prediction error in order to generate higher-order agency judgments. Here, we now extend this prior research on self-agency to another level^1,2,27^ by demonstrating that the mPFC represents one neural site that is able to causally modulate the gain of this prediction error (i.e., increase subjects’ confidence in their internal self-predictions about their speech outcome), to induce subjects to make smaller corrective responses. These smaller rTMS-induced corrective responses predicted improved conscious judgments of self-agency during reality monitoring. Together, the present findings delineate the causal impact of how modulating mPFC activity impacts self-agency, driven by improved reliance on self-prediction mechanisms.

The specific mPFC site we target with high-frequency rTMS is based on our convergent functional imaging studies (across fMRI and MEG imaging), in which subjects showed increased activity within a specific anatomically and functionally consistent mPFC region that mediated self-agency^6,8,20^. Furthermore, we also found treatment-induced restoration of increased activity within this same mPFC site, which improved self-agency judgments, even in chronically-ill patients with psychiatric disorders^1,8,29^. Given these prior convergent data that mPFC supports mechanisms underlying self-agency^1,6,8,20,29^, here, we implemented rTMS to examine the causal mechanisms of whether modulating mPFC excitability not only modulates self-agency, but also improves self-prediction mechanisms that lead to the experience of self-agency on two distinct reality and speech monitoring tasks. In our previous rTMS study^20^, after pilot testing different TMS dosages targeting mPFC, we also demonstrated the optimal dosage (i.e., the best combination of rTMS parameters) that yielded maximum comfort for subjects while inducing significant neuromodulatory effects in the targeted mPFC site that we use here in the present application. For example, we found that rTMS protocols targeting mPFC for longer stimulation durations and higher frequencies (e.g. 20 Hz) reduced tolerability ratings but did not further enhance self-agency judgments, compared to 10 Hz rTMS. Cognitive efficacy and safety of targeting the prefrontal cortex with these TMS dosage parameters have also been validated in other studies without a single participant showing side effects^30–32^. In the present study, we also did not observe a single adverse event with the present rTMS dosage parameters. Overall, this is the first causal proof-of-concept study to indicate that high-frequency 10 Hz rTMS targeting mPFC was not only well-tolerated by participants, but also induced significant improvements in self-agency, that was driven by subjects making improved self-predictions during a speech monitoring task, which potentiated improved self-agency judgments on a reality monitoring task.

We also clarify here that we are not stating that the mPFC represents the only neural correlate of self-agency. For example, prior research has shown that increased neural activity in other regions (e.g. motor regions such as the paracentral lobule/supplementary motor area, basal ganglia and cerebellum), have been observed only prior to self-initiated actions (but not externally-perceived actions), leading to the experience of self-agency^14,33–35^. Specifically, neural activity in these regions have been shown to underlie the ability to reliably predict the outcomes of one’s own actions. Such outcome predictions are used in the feedforward control of motor actions like producing speech, and are furnished by forward models that learn to predict the sensory consequences of motor actions. These models receive efference copy of the vocal motor commands sent from motor cortex to the vocal tract articulators and produce outputs that are adjusted to match the actual sensory feedback (e.g. the auditory feedback) resulting from those motor commands. Once learned, these models represent the accumulated knowledge about past speech productions and are used to for making self-predictions of the sensory outcomes (both somatosensory and auditory) expected to result from the current vocal motor commands.

On the other hand, regions such as the posterior temporoparietal cortex which is located at a distant site from the active mPFC rTMS site, and thought to underlie general attention/salience to sensory information but considered to be an independent site outside of the direct self-agency network, represents an ideal control rTMS site^36–38^. Additionally, convergent evidence from our speech monitoring studies and the meta-analyses of action generation studies and brain stimulation studies, all reveal increased neural activity of this temporoparietal site underlying external agency^16,23,39–41^. Further, prior brain stimulation studies provide causal evidence to show that enhancing excitability within this temporoparietal site, induced subjects to enhance judgments of external agency^23^. Thus, we thought that this temporoparietal site provides a good control site, representing the dissociation between self-agency mediated by the mPFC and external agency, mediated by the temporoparietal site. Indeed, as confirmed by our hypotheses, we did not find any significant changes in either corrective responses during speech monitoring, self-agency judgments during reality monitoring, or a significant association between corrective responses with self-agency judgments after participants completed rTMS targeting this posterior parietal site. Overall, these findings indicate that the mPFC is representative of one neural site whose excitation can causally modulate self-agency, and that these effects cannot be attributable to general rTMS effects or practice effects on the task.

### Limitations

Although the present preliminary results showed enhancement of self-agency across both reality-monitoring and speech-monitoring tasks induced by high-frequency 10 Hz rTMS to the mPFC in the active rTMS group, but not for subjects who completed rTMS to the temporoparietal site, they did not yet yield significant group by time interactions in this relatively small sample of healthy participants. Nevertheless, these preliminary findings provide a first step to show that enhancing mPFC excitability by active 10 Hz rTMS induced participants to make smaller corrective responses during minimal pitch-induced perturbations, which predicted improved self-agency judgments on the reality monitoring task. Together, these data provide the first causal evidence for mPFC activity underlying a unitary sense of self-agency that is driven by the basic ability of making reliable self-predictions during a speech monitoring task, which potentiates self-agency judgments on a reality monitoring task. Future research is needed to test whether these effects are sustained in a larger sample size.

In summary, we provide a novel perspective for investigating causal mechanisms underlying self-agency within two distinct speech monitoring and reality monitoring frameworks, based on one’s own self-predictions about the expected outcome of one’s own actions. In this first-of-its-kind multimodal study, we now deliver robust convergent evidence across two different reality and speech monitoring tasks, revealing that the mPFC represents one critical region that improves self-prediction mechanisms that lead to a unitary experience of self-agency. The present preliminary findings provide early suggestions for innovative neural biomarkers and task-based measures for understanding the underlying the neural basis of self-agency, and also provide the first step toward applying precision-medicine guided neuromodulation targets within this specific mPFC site that mediates self-agency. Such precision-medicine approaches in which we use convergent multimodal functional imaging data (across fMRI and MEG studies) as the basis for guiding neuromodulation target sites^6,8,20^, will enable the development of new TMS treatment interventions not only in healthy individuals but also in patients with psychosis-spectrum disorders who show cardinal impairments in self-agency that contribute to severe psychotic symptoms of hallucinations and delusions^1,8^. In conclusion, our findings contribute to a larger body of literature on self-agency, and by specifying here that the mPFC represents a causal neural site that modulates self-agency, the present research creates a path towards developing new neuromodulation treatments interventions to improve self-agency that will be particularly useful for patients with psychosis disorders who exhibit severe impairments in self-agency.

## Data Availability

The data will be published in the NIMH Data Archive (NDA), as part of the NIMH Data Submission Agreement (DSA) (https://nda.nih.gov/).
